# Proteomic profiling of host-biofilm interactions in an oral infection model resembling the periodontal pocket

**DOI:** 10.1038/srep15999

**Published:** 2015-11-03

**Authors:** Kai Bao, Georgios N. Belibasakis, Nathalie Selevsek, Jonas Grossmann, Nagihan Bostanci

**Affiliations:** 1 Oral Translational Research, Institute for Oral Biology, Center of Dental Medicine, University of Zürich, Zürich, Switzerland; 2 Oral Microbiology and Immunology, Institute for Oral Biology, Center of Dental Medicine, University of Zürich, Zürich, Switzerland; 3 Functional Genomics Center Zürich, University of Zürich, Zürich, Switzerland

## Abstract

Periodontal infections cause inflammatory destruction of the tooth supporting tissues. We recently developed a dynamic, *in vitro* periodontal organotypic tissue model in a perfusion bioreactor system, in co-culture with an 11-species subgingival biofilm, which may recapitulate early events during the establishment of periodontal infections. This study aimed to characterize the global proteome regulations in this host-biofilm interaction model. Semi-quantitative shotgun proteomics were applied for protein identification and quantification in the co-culture supernatants (human and bacterial) and the biofilm lysates (bacterial). A total of 896 and 3363 proteins were identified as secreted in the supernatant and expressed in the biofilm lysate, respectively. Enriched gene ontology analysis revealed that the regulated secreted human tissue proteins were related to processes of cytoskeletal rearrangement, stress responses, apoptosis, and antigen presentation, all of which are commensurate with deregulated host responses. Most secreted bacterial biofilm proteins derived from their cytoplasmic domain. In the presence of the tissue, the levels of *Fusobacterium nucleatum, Actinomyces oris* and *Campylobacter rectus* proteins were significantly regulated. The functions of the up-regulated intracellular (biofilm lysate) proteins were associated with cytokinesis. In conclusion, the proteomic overview of regulated pathways in this host-biofilm interaction model provides insights to the early events of periodontal pathogenesis.

Periodontal infections are the primary reasons for adult tooth loss, due to the destruction of tooth-supporting (periodontal) tissues^1^. While environmental pressures and genetic variations may exert different susceptibilities among individuals on the development of the diseases^2^, these diseases are of inflammatory pathogenesis and initiated by the formation of a microbial biofilm (commonly known as “dental plaque”) which accumulates on the tooth surface^2^. Biofilms are complex polymicrobial communities of endogenous oral species, with more than 700 microbial species already having been identified in the oral cavity potentially constituting part of each biofilm^3^. When in the dense yet dynamic structure of a biofilm, oral microorganisms display increased resistance to environmental stresses^3^. There are structural and functional relationships between the constituent species of the biofilm and the attached surface^4^, which may be symbiotic^5^ or antagonistic^6^. Biofilms consisting of commensal species may actually provide a health benefit to the gingival tissue, by positively priming the immune system^7^. Under certain environmental pressures, a shift of the flora may be favoured towards a more to pathogenic one, triggering disease^1^,^8^,^9^. These concepts are accepted today as a model of polymicrobial synergy and dysbiosis^9^.

The periodontium is a syncytium of specialised tissues that surround and support the teeth. Gingival epithelia line the surfaces of the periodontium and are the first layers that encounter the tooth. They act as physical barriers against infection and aid the immune response with their enzyme-rich lysosomes and semi-permeability that allows the trafficking of immune cells to the site of infection^10^. The underlying connective tissues supports and regulates the function of the epithelium^11^, while protecting the underlying alveolar bones and periodontal ligament that hold the tooth in place. The epithelium and connective tissue are collectively known as the gingival tissue. Periodontal inflammation in response to the causative biofilm is initiated in the gingival tissue^2^. As a result, immune cells, such as polymorphonuclear leukocytes, macrophages and lymphocytes are recruited to the region in order to tackle the establishing bacterial infection. Nevertheless, an excessive and deregulated inflammatory reaction will eventually cause connective tissue breakdown and alveolar bone loss, manifesting as periodontitis^12^.

Periodontal infections are complicated processes that require the understanding of both the microbial and host component, as well as their interaction. Therefore, we recently developed a complex periodontal infection model^13^ that includes an 11-species biofilm used to challenge a generated organotypic tissue consisting of gingival epithelial, gingival fibroblast and monocytic cells grown on collagen sponges. The whole co-culture was performed in a closed dynamic perfusion bioreactor system that ensured the establishment of continuous sheer forces. During that study we also characterized the model morphologically, in terms of cytokine production and bacterial profiling, and concluded that it closely resembles the *in vivo* environment.

The study of periodontal disease on the proteomic level has become increasingly popular in recent years, as a result of improvements in mass spectrometry-based technologies^14^. This has enabled high-throughput clinical studies of protein expression in gingival cervical fluid (GCF), for example^14^. *In vitro* studies on the proteome of cells^15^ and multispecies biofilms^16^,^17^ or host-biofilm interaction infection models^15^ are also available, all of which broaden our knowledge of periodontal infections. Hence, the present study utilised semi-quantitative proteomics with the aim to characterise changes in the proteome that take place in our recently established *in vitro* periodontal infection model^13^. This approach aspires to unravel in more detail the intricate interactions between the gingival tissue and subgingival biofilms during the early stages of the establishment of periodontal inflammation.

## Results

### Qualitative and semi-quantitative proteomic experiments

After 24 h of co-culture of the *in vitro* multispecies biofilm with the organotypic gingival tissue in a perfusion bioreactor^13^, the culture supernatants were collected and processed for proteomic analysis. The following groups were included: a) biofilm-challenged organotypic tissue (tissue + biofilm) b) organotypic tissue alone (tissue) and c) biofilm alone (biofilm). The detailed structural characterization of this multispecies biofilm and organotypic tissue was reported recently^13^. In brief, histology and electron microscopy revealed that the tissue consisted of a superficial epithelial-like layer and an underlying collagen-supported connective tissue rich in gingival fibroblasts and monocytic cells. In presence of the tissue, the growth of selected biofilm species (*Campylobacter rectus*, *Actinomyces oris*, *Streptococcus anginosus*, *Veillonella dispar*, and *Porphyromonas gingivalis*) was suppressed, potentially demonstrating an antimicrobial effect by the tissue.

**Figure 1:**
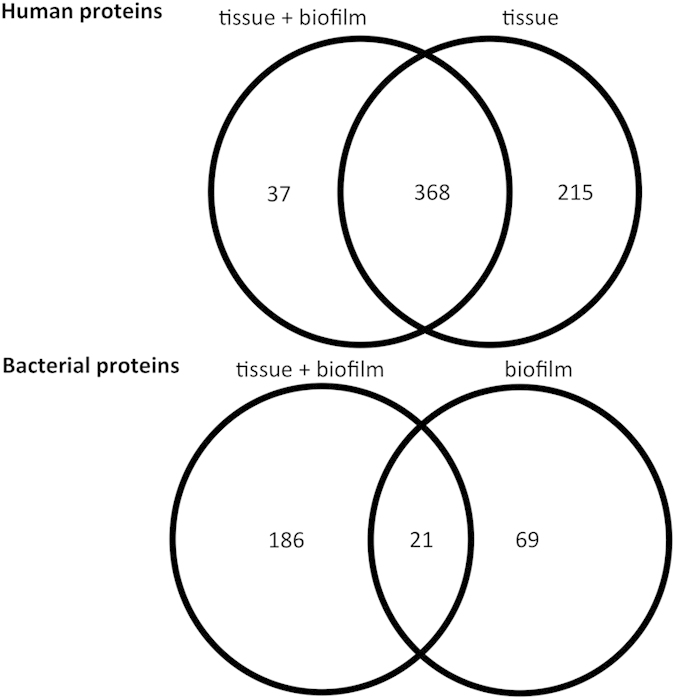
Venn diagram of identified protein numbers in culture supernatants. Protein numbers for each category is depicted. Details of human and bacterial proteins in each group are listed in Supplementary Information Table S1 and Table S2, respectively.

For the proteomic analyses performed in this study, proteins were accepted if at least two peptides were present, with peptide false discovery rate (pepFDR) set to less than 1%, and protein false discovery rate (proFDR) to less than 3%. In this experiment, actual pepFDR and proFDR were 0.65% and 2.4%, respectively. The overlaps of detected human and bacterial proteins in pair-wise comparisons of these three groups are shown in Fig. 1, whereas lists of identified human and bacterial proteins are presented in supplementary Table S1 and supplementary Table S2, respectively. In all, a total of 620 human proteins and 276 bacterial proteins were identified. When human proteins were taken into account, 37 proteins were exclusively identified in the tissue + biofilm group, whereas 215 proteins were exclusively identified in the tissue group alone. There was also a considerable protein overlap between the two groups (n = 368), as shown by the intersection sets of the Venn diagrams (Fig. 1). Of 276 secreted bacterial proteins, 179 were derived uniquely from *F. nucleatum*. Numerically, more proteins belonging to *S. oralis*, *S. anginosus,* and *V. dispar* were identified in the biofilm group, while more proteins belonging to *P. intermedia* were present in the tissue + biofilm group. The species-specific taxonomy of the all identified bacterial proteins numbers is provided in Table 1.

Quantitative differences in protein expression were assessed by spectral counting, as detailed in the methods section. The quantitative comparison was based on proteins identified with *p* value of log_2_ Fold Change (logFC) smaller than 0.05 for the statistical test. With these criteria, a total of 228 proteins of either human (n = 76) or bacterial (n = 152) origin were filtered as significantly regulated ones. For the regulated secreted human proteins, the down-regulated proteins outnumbered the up-regulated ones by approximately 20%. For the regulated secreted bacterial proteins, the up-regulated proteins outnumbered the down-regulated ones by approximately 18%. For the regulated biofilm lysate proteins, the down-regulated ones outnumbered the up-regulated ones by approximately 92%. To graphically represent these data, a volcano plot was used (Supplementary Fig. S1a and Supplementary Fig. S1b).

### Secreted proteins of human origin

**Figure 2:**
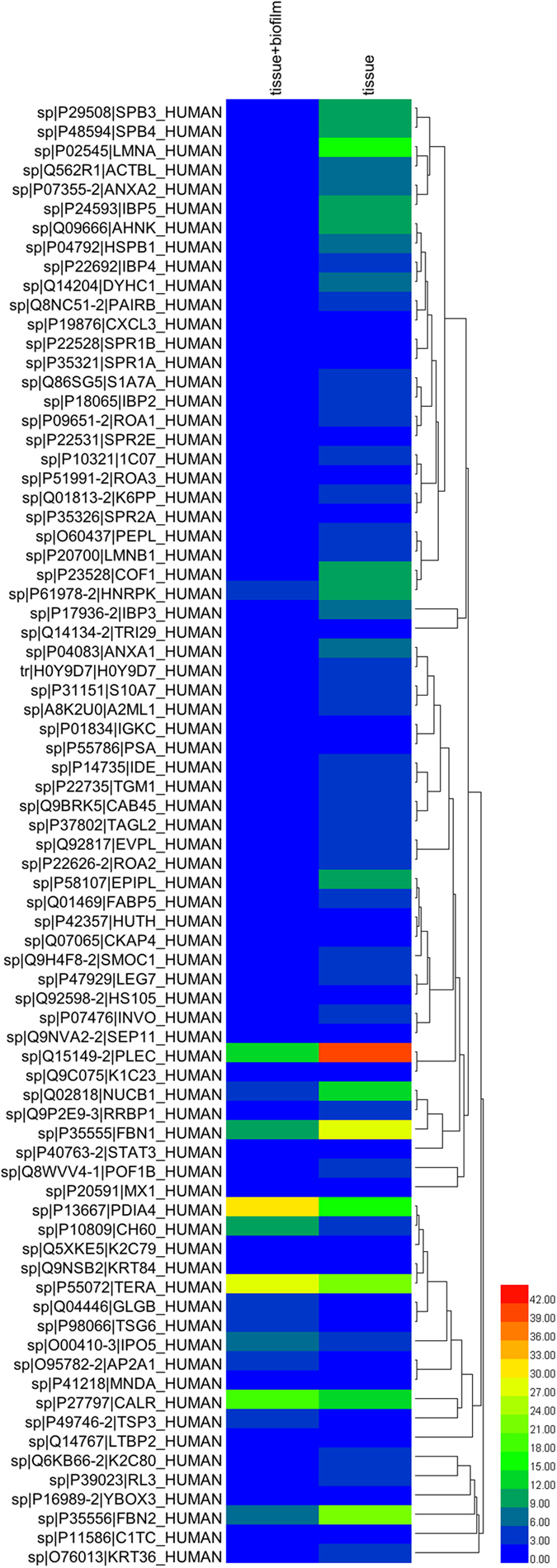
Heat map of differentially regulated human proteins in culture supernatants. Hierarchical clustering of significantly altered proteins (LogFC P<0.05) were performed by the average linkage method using the Heml software. The colours in the map display the mean value for individual proteins (represented by a single row) within each experimental group (represented by a single column). Expression values are shown as a colour scale, with higher values represented by red and lower represented by blue. The colour scale bar gradient is shown at the right corner of the figure.

Out of 76 human proteins, 4 and 54 proteins were uniquely quantified in the tissue + biofilm and tissue groups, respectively, while in the remaining proteins, 18 were jointly quantified in both groups. Of those 18 proteins, 9 were up-regulated and 9 down-regulated in the tissue + biofilm group according to the regulation criteria. The relative enrichment pattern for human proteins is also shown as a heatmap (Fig. 2).

These differentially expressed human proteins (combining both up-regulated and down-regulated ones) were further enriched using the MetaCore software (https://portal.genego.com, Thomson Reuters) following enrichment analysis as described in the methods^18^. The lists of top 10 GO processes and process networks for these proteins in the tissue + biofilm, compared to the tissue group alone are provided in Table 2 and 3, respectively. Regulated proteins in each category are shown in supplementary Table S3. The results were ranked according to increasing *P* value. With respect to GO processes, the most regulated ones involved epidermis and skin development, and also antigen presentation and processing (Table 2). Subsequently, the most over-represented process networks were *cytoskeleton*—*intermediate filaments* (7 out of 81 proteins, *P* value 1.433E-06), *protein folding* (5 out of 69 proteins, *P* value 1.207E-04), and *immune response—phagosome in antigen presentation* (7 out of 243 proteins, *P* value 1.555E-03) (Table 3).

**Figure 3:**
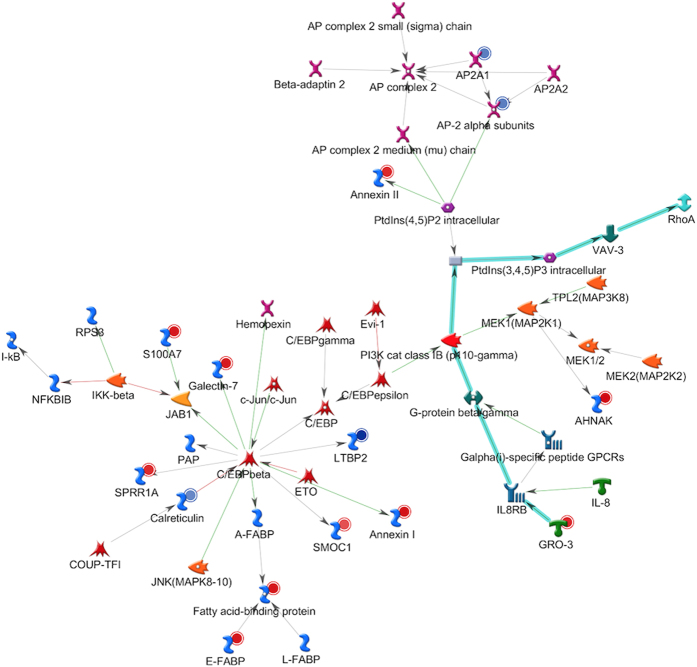
Analysis of the most significantly regulated network: relationships of the regulated human proteins. Networks of protein interactions in regulated human proteins (including Calreticulin, AP-2 alpha subunits, AHNAK, LTBP2, SPRR1A) using MetaCore. The networks between the regulated proteins were calculated based on the “analyse network” algorithm value, then the network maps of their putative protein interactions and related proteins were predicated accordingly from MetaCore database. The regulated human proteins from the list (Table S3) are denoted in smaller circles. Smaller red and blue circles indicated up-regulated and down-regulated proteins, respectively. Green arrows indicate activation of the corresponding proteins; red arrows, inhibition; and gray arrows, unspecified. The cyan highlighted edges in the legend represent the canonical pathways, as recorded by MetaCore. For detailed network symbol legend, see supplementary Figure S4 for full annotations of nodes.

**Figure 4:**
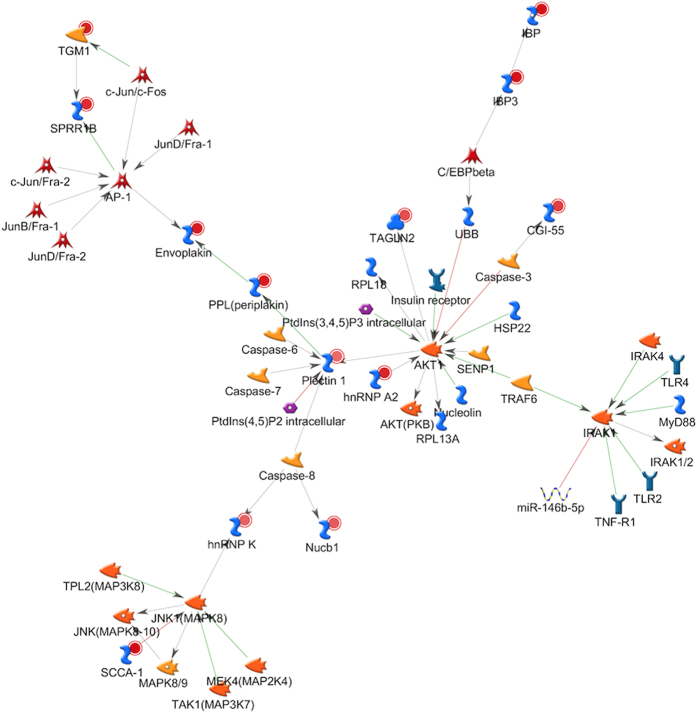
Analysis of the second most significantly regulated network: relationships of the regulated human proteins. Networks of protein interactions in regulated proteins (including Plectin 1, hnRNP K, Envoplakin, TGM1, SPRR1B) using MetaCore. The networks between regulated proteins were calculated based on the “analyze network” algorithm value, then the network maps of their putative protein interactions and related proteins were predicated accordingly from the MetaCore database. The regulated human proteins from our list (Table S3) are denoted in smaller circles. Smaller red and blue circles indicated up-regulated and down-regulated proteins, respectively. Green arrows indicate activation of the corresponding proteins; red arrows, inhibition; and gray arrows, unspecified. For detailed network symbol legend, see Supplementary Figure S4 for full annotations of nodes.

**Figure 5:**
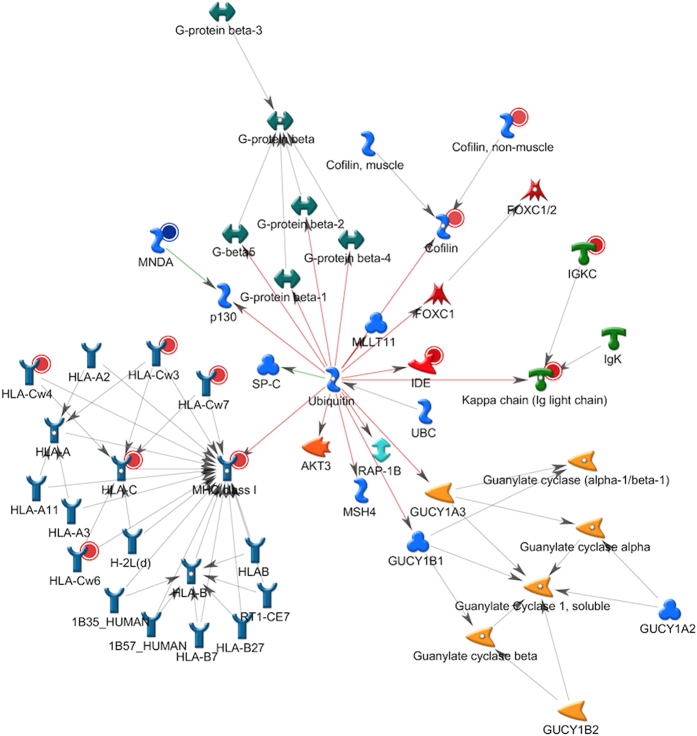
Analysis of the third most significantly regulated network: relationships of the regulated human proteins. Networks of protein interactions in regulated human proteins (including IDE, Kappa chain (Ig light chain), Cofilin, MNDA, MHC class I proteins) using MetaCore. The networks between regulated proteins were calculated based on the “analyze network” algorithm value, then the network maps of their putative protein interactions and related proteins were predicated accordingly from the MetaCore database. The regulated human proteins from our list (Table S3) are denoted in smaller circles. Smaller red and blue circles indicated up-regulated and down-regulated proteins, respectively. Green arrows indicate activation of the corresponding proteins; red arrows, inhibition; and gray arrows, unspecified. For detailed network symbol legend, see Supplementary Figure S4 for full annotations of nodes.

A network built using the “analyse network” algorithm to connect the proteins in this process. The top 10 most predominant potential protein interaction networks among these regulated human proteins associated with all involved GO processes and given *P* value are listed in Table S4, while the regulation relations of the top 3 groups are highlighted in Figs 3, 4, 5. The first top regulated network named *Calreticulin, AP-2 alpha subunits, AHNAK, LTBP2, SPRR1A* revealed activation of damage-associated molecular patterns, including calreticulin and down regulation of cell-adhesion molecules such as latent transforming growth factor beta binding protein 2 (Fig. 3). This network has a total of 51 proteins including 14 of them from our regulated list. The *P* value of the network is 8.89E-32, which indicated that the probability of assembly from a random set of nodes (proteins) was very low (see supplementary Table S4). *Regulation of response to stress*, *cellular response to chemical stimulus, regulation of defence response*, and *immune system process* were among the most associated GO terms suggesting that these proteins may be involved in the initiation of periodontal infection. Notably, within this network, a chemokine pathway- GRO-3-IL8RB-Actin cytoskeletal—including GRO-3, and interleukin (IL)-8 receptor was present (canonical pathway, cyan colored interaction arrows). While IL-8 has been extensively studied in periodontal infection, little is known about GRO-3 although it has capacity to activate neutrophils.

The second top regulated network named *Plectin 1, hnRNP K, Envoplakin, TGM1, SPRR1B* (Fig. 4) constituted 50 proteins, including 13 proteins from our regulated list with a *P* value of 4.3E-29. Further analysis of the proteins in the network suggested that disease associated biofilm challenge caused highly coordinated activation of several proteins associated with toll-like receptor pathways. The third most affected network named *IDE*, *Kappa chain (Ig light chain), Cofilin, MNDA, MHC class I* featured activation of antigen processing and presenting pathways (Fig. 5).

Furthermore, mapping of the regulated proteins onto canonical pathway maps revealed that most altered pathways including *cytoskeleton remodelling-filaments*, *regulation of degradation of deltaF508-CFTR in CF* and *IL-13 signaling via JAK-STAT.* Intracellular filaments including epithelial keratins are mainly located in the cytoplasm and nucleus and are cross-linked by binding proteins like plectin, envoplakin, periplakin, epiplakin. These proteins are involved in maintaining cell and tissue integrity. IL-13 is known to play an important role in the regulation of immune responses and is also implicated in other pathological conditions, but not yet in periodontal infection. This indicates alterations in cellular integrity and immunity, in response to disease-associated biofilms.

### Secreted proteins of bacterial biofilm origin

**Figure 6:**
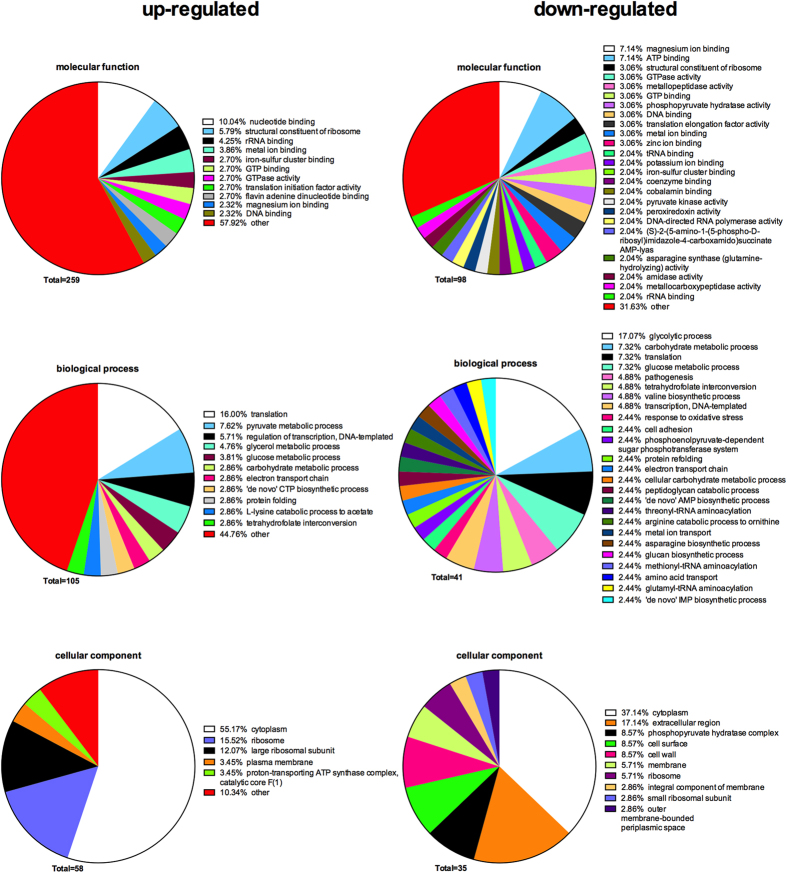
Annotation of regulated secreted bacterial protein functions by Gene Ontology (GO) terms enrichment. The GO terms from all regulated bacterial functions were categorized into biological process, molecular function, and cellular component as displayed in pie charts use tissue + biofilm group, compared to the biofilm group alone. The numbers of GO terms for each of the three categories are shown, whereas the proportion of each specific subcategory is also provided. Subcategories with GO terms less than 2% are classified as “other”.

For secreted bacterial proteins, the regulated trends are shown in Table 4. Among the significantly regulated proteins, 128 were increased (up-regulated, positive Log FC value), whereas 24 were decreased (down-regulated, negative Log FC value) in the biofilm-challenged tissue group versus the un-challenged tissue group. The functions of the significantly altered bacterial proteins were manually enriched by GO process terms, according to Uniprot (Fig. 6). In general, a total of 259, 105, and 58 GO terms were generated for molecular function, biological process, and cellular component, respectively, based upon the up-regulated proteins in the tissue + biofilm group, compared to the biofilm group alone. Among the down-regulated proteins, 98, 41, and 35 enriched GO terms corresponded to molecular function, biological process, and cellular component, respectively. In general, less GO terms appeared for up-regulated, compared to down-regulated proteins. Besides, *cytoplasm* was the most common enriched cellular component GO term, in both down regulated and up-regulated proteins (37% and 55% of all terms, respectively). Finally, 17% of the cell component GO terms in down-regulated proteins came from extracellular region, and this percentage was not matched in the up-regulated proteins.

### Intracellular protein changes of biofilms in response to the host tissue

As also described earlier^13^, upon completion of the experimental period, the biofilms were able to survive, although most of the bacterial species were suppressed in the presence of the organotypic host tissue. To evaluate such proteomic changes within the subgingival biofilms their cell lysates were collected and processed for further proteomic analysis. In this experiment, the actual pepFDR and proFDR were 0.2% and 1%, respectively.

**Figure 7:**
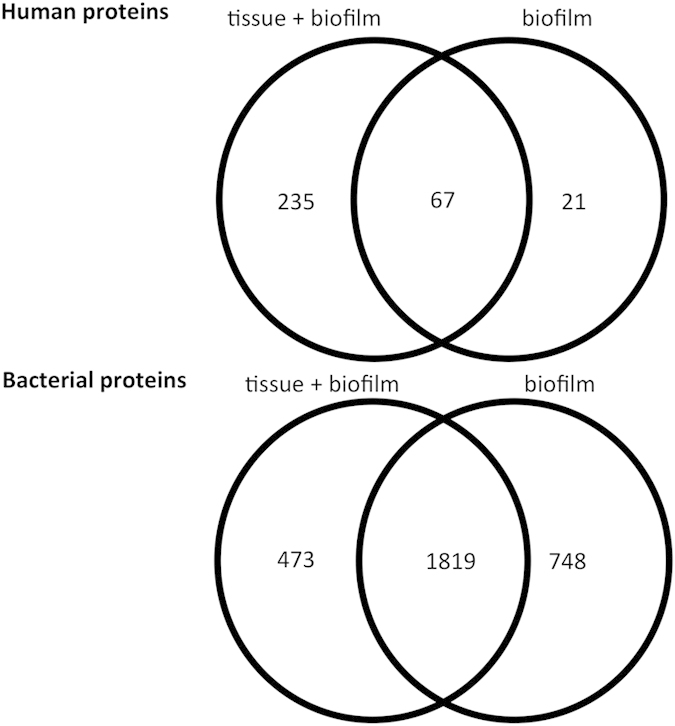
Venn diagram of identified protein numbers in biofilm lysates. Protein numbers for each category is depicted. Details of proteins in each group are listed in Supplementary Information Table S5.

In the tissue + biofilm group, a total of 2594 proteins were identified (302 human, and 2292 bacterial proteins). In the biofilm alone group, a total of 2655 proteins were identified (88 human and 2567 bacterial). The overlaps of detected proteins between these two groups are shown in Fig. 7. The species-specific taxonomy of the identified protein numbers are provided in Table 5, whereas Supplementary Table S5 presents the detailed information of total unique peptide counts and the annotation of proteins identified the biofilm lysates. Interestingly, 202 proteins of *S. oralis* were uniquely identified in the organotypic tissue group, while only 3 such proteins were uniquely identified in the biofilm group. Conversely, only 1 protein of *V. dispar* was uniquely identified in the tissue + biofilm group compared with 371 proteins in the biofilm group. Of note, only 2 proteins from *T. forsythia* were identified in total. Given the numerically low amount of *T. forsythia* in the biofilm^13^, this might give an underrepresentation of proteins in the biofilm.

To further evaluate the abundance of the identified proteins, spectral counting was used to further compare the proteomic expression profiles in the biofilm lysates. The list of identified proteins is presented in Supplementary Table S5. The human proteins identified in these lysates could be due to false discovery, or constitute remnants of the biofilm growth medium (which contained human serum), or of the hydroxyapatite-coating salivary pellicle on which the biofilms were grown. Nevertheless, these were not further considered, in order to maintain the focus on the regulated bacterial proteins. A visualized result of regulated proteins among all proteins is shown in a volcano plot (Supplementary Fig. S1c), using the -1*log_10_ (P value) vs. log_2_ (fold-change ratio). Hence, a total of 1081 proteins were classified as regulated proteins. Of those, 518 proteins were up-regulated, whereas 563 proteins were down-regulated (Table 6) in the tissue + biofilm group, compared to the biofilm alone. Interestingly, proteins from each species showed different tendencies of regulation. For example, all regulated proteins of *V. dispar* in the tissue + biofilm group were down-regulated compared to the biofilm group alone whilst *A. oris* and *T. denticola* only had up-regulated proteins. When the *V. dispar* proteins were ranked according to their relative significant abundance, among the top five regulated ones were carboxylase domain protein, phosphoglycerate kinase, pyruvate synthase, methylmalonyl-CoA mutase and formate C-acetyltransferase. The top five up-regulated *T. denticola* proteins were oligopeptide/dipeptide ABC transporter periplasmic peptide-binding protein, filament protein A, lipoprotein, OppA protein and major outer sheath protein.

**Figure 8:**
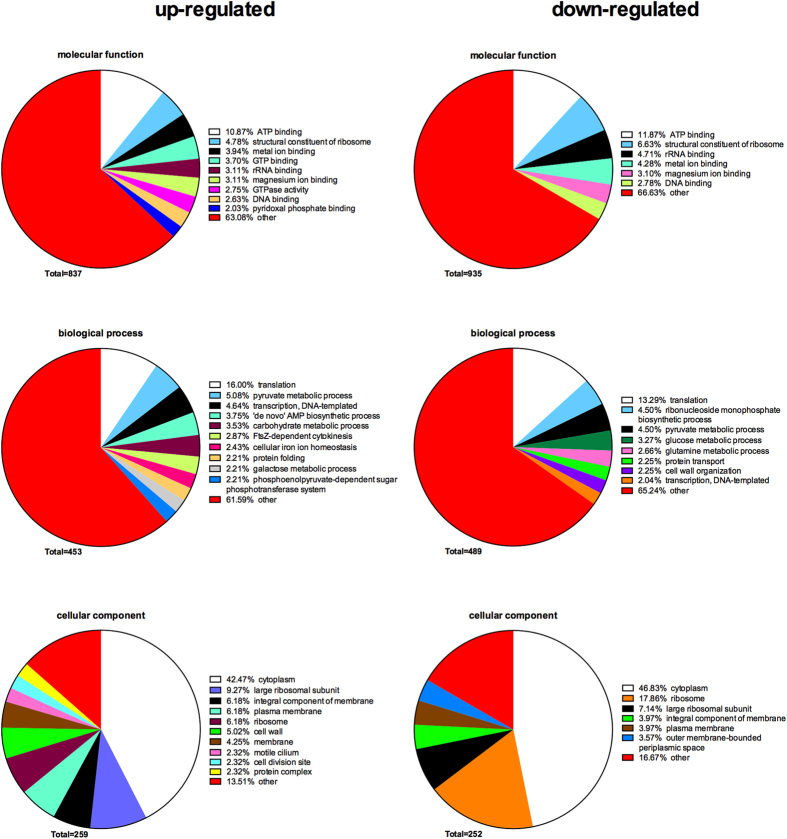
Annotation of regulated bacterial protein functions in biofilm lysates by enrichment of Gene Ontology (GO) terms. The GO terms from all regulated bacterial functions were categorized into biological process, molecular function, and cellular component as displayed in pie charts use tissue + biofilm group, compared to the biofilm group alone. The numbers of GO terms for each of the three categories are shown, whereas the proportion of each specific subcategory is also provided. Subcategories with GO terms less than 2% are classified as “other”.

The GO term enrichment from regulated bacterial proteins is shown as a pie chart (Fig. 8). A total of 837, 453, and 259 GO terms were generated for molecular function, biological process, and cellular component, respectively, based upon the up-regulated proteins in the tissue + biofilm, compared to the biofilm alone. For the down-regulated proteins, a total of 935, 489, and 252 GO terms were generated. Among the enriched GO terms of molecular function, “ATP binding” was the most common one in both up-regulated (13%) and down-regulated (11%) groups. “Translation”, constituted 16% and 13% of the most common enriched up-regulated and down-regulated GO term for biological processes, respectively, Regarding cellular localisation, more than 40% of up- and down-regulated proteins derived from the “cytoplasm” domain. However, some unique functions also appeared in each of these three categories. For molecular functions, the GO terms of *GTP binding* and *GTPase activity* were restricted only to the up-regulated proteins. For biological process, *FtsZ-depend cytokinesis* was unique among up-regulated proteins. Finally, for cellular component, 2% of GO terms came from *cell division site*, which appeared only in up-regulation.

## Discussion

Considering the biological resemblance to the *in vivo* environment, its feasibility to culture and its relatively low cost compared to other *in vivo* models, the mucosal organotypic models^19^,^20^ in conjunction with a multispecies biofilm^13^,^15^,^21^,^22^ are becoming increasingly utilised for studying the interactions between host tissues and oral biofilms. However, few of them combine both these elements together for studying infection processes of periodontal disease^15^. Our recently developed organotypic model, cultivating an epithelial-fibroblast and monocyte containing tissue in a three dimensional conformation, which was thereafter challenged by a multi-species biofilm in a perfusion bioreactor, is one of the most complex infection systems currently available^13^. In the present study, we characterized the full secreted proteome (‘*periodontal exudatome*’) of this recently described model, which includes the integrated analysis of both host tissue and biofilm bacteria secreted proteins. We also characterized the proteomic changes occurring in the biofilm itself when in contact with the tissue, by analysing lysates of its cell-associated content. First, we used label-free bottom-up proteomic technology to screen and identify target proteins that were regulated in the gingival extracellular milleu upon interaction with disease associated biofilms. Second, we generated functional gene networks and pathway maps using the Metacore tool in order to integrate the interactions of the identified human proteins. The combination of mass-spectrometry based label-free quantitative proteomics with bioinformatics analysis provided extensive insights into understanding the host-biofilm interaction dynamics in a given microenvironment. This approach provides a holistic view of the early events that may take place in the gingival tissue as the associated biofilms, during the initiation of periodontal inflammation.

One of the first qualitative observations in this study was that more secreted human proteins were detectable in the tissue when biofilms were absent. On the contrary, under biofilm challenge, fewer proteins were detectable and the most were down-regulated compared to the control. This supports the notion that biofilm challenge can dampen the host responses to favour microbial survival and establishment in the tissue^23^. It is also supported by previous reports in gingival organotypic epithelial cultures studying either the whole secretome^15^ or individual cytokines^24^, and reports in gingival fibroblasts demonstrating that biofilms down-regulate key intracellular components of the inflammatory response^25^,^26^. The present organotypic tissue model contains both the epithelial and connective tissue component of gingiva, as well as cells of the immune system, and further reinforces these earlier works.

The collected supernatant of the bioreactor culture was used to analyse the ‘*periodontal exudatome*’ in this model. The content of this supernatant may share some similarities to the gingival cervical fluid (GCF) *in vivo*. Indeed, some of the identified proteins, especially those associated with immune responses, including annexin A1, calgranulin B, cathepsin G, were also found in previous proteomics analysis performed in GCF^27^. Although the other identified proteins, like HSP60, HLA-C were not readily identified in the proteomic studies of GCF, earlier evidence has shown that these proteins can exist in periodontal tissues^28^.

In our recent work we reported the morphological disruption of the epithelium-like surfaces on this organotypic tissue when co-cultured with biofilms^13^. The bioinformatics analysis of the regulated proteins performed here identified that cytoskeletal remodelling, keratinisation and deregulations in protein unfolding were among the most affected process networks, whereas epidermis development and skin development were among the most affected GO processes. Hence, the morphological observations of epithelial disruption concur with the regulated process on the proteomic level. While healthy and minimally challenged, the oral epithelium exhibits a rapid turn-over in order to maintain a tolerable host-bacterial equilibrium^29^, a process that is impaired in the course of periodontal infection.

Disruption of epithelial integrity could also be explained by regulation of lamin A/C and lamin B1 as found in this experimental model. Lamins are components of the inner nuclear member, believed to play an important role for nuclear assembly and chromatin organisation. They are also related to the many apoptosis signalling pathways^30^, which also ranked among the top regulated process networks in response to the biofilm challenge. Considering that lamins are mainly found in cytoplasm, increasing their presence in the bioreactor supernatant probably originates from lysed epithelial cells, which could also be interpreted as cell death. In line with apoptosis, negative regulation of cell proliferation was also one of the enriched process networks. Interestingly, lamin A/C is known to effect osteogenesis^31^, which could potentially be involved in alveolar bone resorption occurring at later stages of periodontal infection. Together, all these processes indicate that the presence of the biofilm affected tissue growth, induced apoptosis and consequently led to it structural disruption, as previously observed microscopically^13^.

Apart from the physical barrier to pathogens, epithelium is known to mediate the innate immune responses by recruiting cells of the immune system, such as neutrophils and antigen presentation to the T-helper cell^32^. The bioinformatic enrichment of GO processes revealed the regulation of *antigen processing and presentation* of endogenous and exogenous peptide antigens via MHC class I. Normally, MHC class I are considered to be regulated by viral peptides, although bacteria are also found to manipulate these pathways^33^. Immune IL-13 signalling was also one of the top 10 regulated process networks. As part of its role in inflammation roles, IL-13 induces the production of matrix metalloproteinases (MMPs)^34^. Since MMPs are important molecules to degrade primarily the connective tissues^35^, this finding could be well in line with the disruptions of gingival fibroblast attachment, described in our previous report^13^.

Finally, GO processes related to toll-like receptors (TLRs) appeared in 3 out of top 10 enriched network links of regulated proteins in bioreactor supernatant (Table S4). Although TLRs are potentially expressed in all three host cell types represented in our models, they might have different functions during the processes of periodontal infections. For epithelial cells, TLR is important to recognise different bacterial Pathogen-Associated Molecular Patterns (PAMPs)^36^, and maintain the balance between commensal bacteria and epithelial integrity through a well-controlled innate immune response. *P. gingivalis*, a constituent species of the present biofilm model, may avoid the activation of TLRs through its cell surface fimbriae (FimA), which may however not evade detection by monocytes^37^. In the case of fibroblasts, following epithelial layer degradation, increasing TLR stimulation may result in the expression of inflammatory factors that further stimulate tissue degradation^38^. Activating these TLR pathways is also well in line with the increased cytokine expression, as previously reported^13^.

Biofilms are the initiating factor of periodontal infections. Secreted bacterial proteins from the biofilms may regulate host tissue functions in a manner that is detrimental for the pathogenesis of periodontal disease^39^,^40^. Conversely, dynamic changes are also imposed on the microbiological profile of the biofilms during their interaction with host tissues, a concept well summarized by the ‘ecological plaque hypothesis’^41^,^42^. In the present biofilm model we hold the technical advantage of knowing in advance the specific constituent species, and therefore we could manually enrich the GO terms of all the bacterial proteins ascribed to the function of the whole biofilm^17^ to avoid the low consistency in *in vivo*. The number of identified secreted bacterial proteins was less than half (44%) the number of human proteins. However, only 7% of these proteins were identified in biofilm lysates under both tissue challenged and biofilm alone conditions. This left a large amount of them to be considered as “regulated proteins”, as they were absent from the occasional comparison group. Interestingly, *F. nucleatum* expressed more than half of both identified and regulated proteins. Moreover, these numbers in the biofilm-challenged tissue group were higher than the biofilm alone group, despite the lower (but not significantly) bacterial cell numbers. In terms of biofilm formation, *F. nucleatum* plays an important role in connecting early colonizes with late colonizes due to its ability that co-aggregated with many different species^43^. Besides, it is also reported that *F. nucleatum* can invade oral epithelial^44^ and fibroblastic cells^45^, and its culture filtrates strongly induced apoptosis of monocytic cell lines^46^. All these functions may explain the relatively stable numbers of *F. nucleatum* proteins in the presence of the organotypic tissue. Moreover, this bacterium has also been reported to increase the virulence^47^ and attachment to the host cells^48^ of *P. gingivalis*. Therefore, as one of the most abundant gram-negative species^43^, *F. nucleatum* may have a mechanistic advantage in surviving the host challenge, while maintaining a relatively stable bacterial number.

For further bioinformatics analysis of bacterial proteins in the bioreactor supernatant, the biofilm was considered as a whole unit, rather than by its individual species. Hence, the pie charts of enriched GO terms of all the bacterial proteins were divided into three separate ontologies: 1) molecular function, 2) biological process, 3) cell component. The most popular enriched GO term from molecular function category among up-regulated bacterial proteins in the tissue + biofilm group was *nucleotide binding*. This result is consistent with the observation that some of the most popular enriched GO term in the biological process category were *translation* and *protein folding*. Another interesting finding is that the second popular GO term among the down-regulated bacterial proteins in the cell component category is the *extracellular region*, which was not prevalent among the up-regulated proteins. This may indicate the presence of more intact bacteria in the biofilm alone group.

Intracellular proteins of the biofilm lysate were further considered. More proteins were identified in biofilm lysates compared to the secreted bioreactor supernatants. More interestingly, although the tissue-encountered biofilms synthesized significantly less intracelluallar bacterial proteins, most of them were regulated, reflecting an adaptational response to the tissue. In fact, different patterns of proteome adaptation were observed for the individual bacterial species. Unlike in the bioreactor supernatant, intracellular *F. nucleatum* proteins did not show a strong regulation in the presence of the organotypic tissue. Instead, although present at lower cell numbers, *Actinomyces oris* and *Campylobacter rectus* displayed more identified and regulated proteins in the biofilm lysates, in the presence of the organotypic tissue. Considering that the numbers of these two species was also lower in the tissue + biofilm group, this trend was less likely caused by different protein input. *A. oris* is an early colonizer, reported to have a mutualistic growth with *P. gingivalis*^49^. In the biofilm, relationships between *A. oris* and *Streptococcus spp*. are associated to co-aggregation^43^ and possible the regulation of *Streptococci spp*. through digestion of quorum sensing molecule autoinducer 2^50^. Lack of *A. oris* and *Streptococcus* spp. from this “subgingival” biofilm model is clearly shown to impose a number for structural and compositional changes in the rest of the biofilm species^51^. *C. rectus*, appears in relative late stage of biofilm formation^52^ and is clinically associated with periodontal infections. In *in vivo* studies, *C. rectus* was found to increase the TLR-4 expression in mice^53^, or cause alveolar bone loss in the capuchin monkey^54^. Both of the streprococci, *A. oris* and *C. rectus* and *T. denticola* appear to increase their cytoplasmic protein expression in the present of the tissue. One prime example of *T. denticola* up-regulated proteins was its major outer sheath protein. This primary virulence determinant in *Treponema denticola,* known to induce cytotoxic responses, inflammatory pathways and inhibit chemotactic events in host cells^55^. These results indicate that, under tissue challenge, the virulence of specific pathogenic bacteria may be enhanced.

The enriched GO terms among regulated proteins showed a trend of cytokinesis of the bacteria in the biofilm, when in presence of the organotypic tissue. Among the categories of biological process, one of the unique GO terms appearing in up-regulated proteins was *filamenting temperature-sensitive mutant Z (FtsZ)-depend cytokinesis*. FtsZ is an essential and highly conserved bacterial cytokinesis protein^56^. Using their C-terminal GTPase-activating domain, FtsZ constructs a Z-ring structure at the site of spectrum formation^57^. This could also explain the appearance of the unique GO term of *GTP binding* and *GTPase activity* among up-regulated proteins in the category of molecular function. Moreover, the cell component category displayed a unique group of proteins identified as *cell division site*. According to the enriched proteins, these may result in thicker cell bacterial walls rendering the bacteria more mobile.

## Conclusion

This study dissected the protein cross-talk between oral biofilms and organotypic gingival tissue, in a dynamic, complex *in vitro* experimental model of periodontal infection. In the presence of the biofilm, less secreted proteins of the organotypic tissue are detectable, implying a down-play of the host responses. Proteomic profiling of the secreted host proteins revealed a tendency for cytoskeletal rearrangement, stress responses, apoptosis and antigen presentation. These are commensurate with disruption of the tissue barrier and activation of immune recognition responses. On the biofilm side, most of the secreted bacterial proteins derived from the cytoplasmic domain, whereas the functions of the up-regulated intracellular proteins were associated with cell division and cytokinesis. These affected host and biofilm pathways may represent early events of periodontal pathogenesis, and should be further investigated in translational studies for their role in the establishment of periodontitis.

## Methods

### Biofilms and bioreactor medium collection

Biofilms and bioreactor medium used in this work were collected from our previous study^13^. Briefly, in this *in vitro* model we co-cultured an 11-species biofilm with gingival organotypic tissue in a perfusion bioreactor system (UCUP, Cellec Biotek AG, www.cellecbiotek.com, UCUP001) to evaluate the effects of biofilm on the tissue. The 11-species biofilm used in this study included the following species: *Prevotella intermedia* ATCC 25611T (OMZ 278), *Aggregatibacter actinomycetemcomitans* JP2 (OMZ 295), *Campylobacter rectus* (OMZ 398), *Veillonella dispar* ATCC 17748T (OMZ 493), *Fusobacterium nucleatum subsp. nucleatum* (OMZ 598), *Streptococcus oralis* SK248 (OMZ 607), *Treponema denticola* ATCC 35405T (OMZ 661), *Actinomyces oris* (OMZ 745), *Streptococcus anginosus* ATCC 9895 (OMZ 871), *Tannerella forsythia* (OMZ 1047) and *Porphyromonas gingivalis* W50 (OMZ 308). Immortalized gingival epithelial cells and gingival fibroblasts, as well as a monocytic cell line^20^ were perfused through 3D collagen sponge (porcine collagen, type I) scaffolds (Optimaix, Matricel GmbH, O3D304030) into the bioreactor to create organotypic tissue. Later, the 11-species biofilm models were co-cultured with the generated organotypic tissues in the bioreactor for 24 h, in order to mimic *in vitro* the gingival tissue-biofilm interface. In parallel, organotypic tissues or biofilms alone, each represented in biological triplicates, were also cultured under the same conditions, serving as controls. The individual bacterial species in the biofilms were quantified as previously described^13^. The medium collected from the bioreactor under each of the 3 different experimental conditions after 24 h, was centrifuged at 1500 rpm for 5 min, and filtered through 0.2 μm pore size. Both medium and biofilm were stored at −80 °C, for the further usage.

### Sample preparations

The collected bioreactor media (i.e. culture supernatants) were subjected to Amicon Ultra-4 cut off 3 kDa (millipore) to concentrate and processed with ProteoMiner Protein Enrichment Kits (Bio-Rad) following the manufacturer’s guidelines to increase the abilities to detect the low-abundance proteins. Proteins lysates of the biofilm were extracted as described previously^17^. Briefly, the biofilms were collected by centrifugation, lysed in medium with 4% w/v Sodium Dodecyl Sulfate (SDS), 0.1 mM dithiothreitol (DTT) and 100 mM Tris-HCl pH 8.2, then sonicated using high intensity focused ultrasound (UTR2000, Hielscher).

### Protein digestion and C18 clean up

After sample preparation, the collected bioreactor media or processed biofilm lysates were measured with Qubit® Protein Assay Kit (Life Technologies). Solutions of 20 μg of proteins per each sample were subjected on the filter device with relative molecular mass cut-offs of 30,000 (30 k filter), for the digestion and detergent remove as described previously^17^. Briefly, the samples were denatured with 8 M urea in 100 mM Tris/HCl buffer (addition 0.1 mM DTT to the bioreactor medium), alkylated with 0.05 M iodoacetamide, and washed by 0.5 M NaCl. They were then digested by trypsin (Promega) at an enzyme to protein ratio of 1:40 in 0.05 M triethylammonium bicarbonate (TEAB) medium in wet-cell chamber overnight. The digested peptides were collected by centrifugation and the reactions were stopped by adding trifluoroacetic acid (TFA) to a final concentration of 0.5%. Digested solutions were desalted using reverse phase cartridges Finisterre SPE C18 (Wicom International AG) as described previously^17^. Briefly, SPE C18 cartridges were first wet with 100% methanol, followed with 60% of acetonitrile (ACN) in 0.1% TFA, equilibrated with 3% of ACN in 0.1% TFA. Then, the digested solutions were loaded onto cartridges, washed with 3% ACN in 0.1% TFA, eluted with 0.5 ml of 60% ACN in 0.1% TFA, dried in vacuum centrifuge, and solubilized with 30 μl 3% ACN in 0.1% formic acid. The desalted samples were stored at −20 °C until further use.

### LC-MS/MS analysis and database search

3 μl desalted samples were then injected into a Q-Exactive mass spectrometer (Thermo Fisher Scientific) for proteomic analysis. The peptides were separated on an Easy nano-flow HPLC system (Thermo Fisher Scientific) coupled to a fused silica emitter (15 cm long, 75 μm diameter) packed with a ReproSil-Pur C18-AQ 120 A and 1.9 μm resin (Dr. Maisch HPLC GmbH). The linear gradient of acetonitrile/water (acetonitrile gradient from 2 to 35% in 120 minutes) with 0.1% formic acid was used to separate peptide at a flow rate of 300 nl/min. A data-dependent method that automatically switches between MS and MS/MS using a top-12 method was used to acquire mass spectrum. These spectra were then acquired in the Orbitrap analyzer at a mass range of 300–1700 *m/z*. The higher energy collisional dissociation (HCD) peptide fragments acquired at 28 normalized collision energy were analyzed at high resolution.

### Protein Identification

Database searches were approached as described previously^17^. Briefly, mass spectrum files were generated using Proteome Discoverer (Thermo) v. 1.4, and then analyzed with Mascot (version 2.4.1) using a customized database consisting of *Homo sapiens* database from Uniprot (release date 22 May 2014, including isoforms) and 11 bacterial species database from NCBI database (release date 28 February 2014). The human database includes 88,708 sequences; the bacterial database includes 228,240 sequences; while known MS contaminants database includes 260 sequences. Same numbers of reverse sequences (ie: 88,708, 228,240, and 260, for human, bacterial, and MS contaminants database) are also included in database as decoy. The precursor and fragment ion tolerance were 10 ppm and ± 0.05 Da, respectively. For tryptic digestion options, up to two missed cleavages per peptide were allowed. Both carbamidomethylation and oxidation option were selected as variable modification parameters. The Mascot research results were imported into Scaffold (version Scaffold_4.2.1, Proteome software) to validate MS/MS-based peptides and protein identifications. The protein list was filtered at a 3.0% protein false discovery rate (protFDR) with 2 minimal peptides, and 1.0% peptide false discovery rate (pepFDR) threshold.

### Spectral count-based label-free quantitation

Spectrum counting was applied to quantify relative protein content. The quantitative values assigned to each identified protein by Scaffold (version scaffold_4.40) and used for calculation of relative protein abundance. The quantitative values were normalised for individual protein expression between different samples by correcting the raw spectral counts by the total number of spectra observed in a given sample. Further calculations were done with R using the EdgeR package^58^. We reported data as log_2_ Fold Change (logFC), log-counts-per-million (logCPM), and related *p* value for each protein between the compared conditions. Proteins were considered to be significantly different in terms of abundance if the *p* value was ≤ 0.05. Furthermore, volcano plots were generated with R to visualize different expression.

### Data visualization by heat map and cluster analysis

The regulated trends of secreted human proteins were visualised by use of heat-map made by Heml (version 1.0.1)^59^. Hierarchical clustering analysis was done with the average linkage method using the Heml software. Quantitative heat map displays the mean quantitative value for each protein, previously calculated with Scaffold software.

### Gene Ontology (GO) analysis of differentially expressed human proteins

To annotate the protein functions at the gene level, differentially expressed human proteins were extrapolated to MetaCore database (released on 14^th^ May 2015) for “enrichment analysis” and “built network”. Through the “enrichment analysis”, Metacore calculated statistically significant enriched “GO processes” and “process networks” based on the probability of GO terms and networks assembly from the regulated proteins among the human protein information in their database as a *p* value. Accordingly, MetaCore is able to provide a quantitative analysis of the top 10 relevant biological functions. To show these proteins in the context of their interacting network, these proteins were further processed to “built network”, using the “analyze network” algorithm, one of the nine network building-algorithms in MetaCore. The *P* values of the resulting network are the possibility of the potential networks according to the curated human protein interaction database within the MetaCore.

### Gene Ontology (GO) analysis of differentially expressed bacterial proteins

We also analysed how the bacterial proteome changes in the bioreactor culture supernatants, as well as in the biofilm lysates^17^, in the presence or absence of the organotypic tissue. Briefly, GO terms from all the regulated bacterial proteins were assembled from the UniProt Knowledgebase release date 14^th^ April 2015) enriched by REVIGO (release date 21^th^ April 2015)^60^, and manually summarized into pie charts. The enriched GO terms were listed from the highest to the lowest according to their proportions among all the terms. The terms that show less than two per cent were clustered into the “other” category.

## Additional Information

**How to cite this article**: Bao, K. *et al.* Proteomic profiling of host-biofilm interactions in an oral infection model resembling the periodontal pocket. *Sci. Rep.* **5**, 15999; doi: 10.1038/srep15999 (2015).

## Supplementary Material

Supplementary Information

Supplementary Dataset 1

## Figures and Tables

**Table 1:** Number of identified secreted bacterial proteins in culture supernatants by each species.

	tissue + biofilm	overlap	biofilm
*A. oris*	1	0	0
*A. actinomycetemcomitans*	1	0	0
*C. rectus*	0	0	1
*F. nucleatum*	168	5	6
*P. gingivalis*	1	0	0
*P. intermedia*	11	3	2
*S. anginosus*	1	4	8
*S. oralis*	3	8	37
*T. forsythia*	0	0	0
*T. denticola*	0	1	0
*V. dispar*	0	0	15

Number of proteins identified from culture supernatants were grouped and listed on the table based on whether they were identified in tissue + biofilm, biofilm alone, or overlap group (identified in both groups).

**Table 2:** Top 10 enriched GO processes of regulated human proteins in culture supernatants.

No.	Processes	Regulated	p-value	FDR
1	epidermis development	15/405	2.081E-11	3.965E-08
2	antigen processing and presentation of endogenous peptide antigen via MHC class I via ER pathway, TAP-independent	6/19	5.517E-11	5.255E-08
3	skin development	15/468	1.575E-10	7.548E-08
4	keratinocyte differentiation	10/146	1.585E-10	7.548E-08
5	antigen processing and presentation of exogenous peptide antigen via MHC class I, TAP-independent	6/25	3.538E-10	1.025E-07
6	antigen processing and presentation of endogenous peptide antigen via MHC class I via ER pathway	6/25	3.538E-10	1.025E-07
7	keratinization	8/76	3.765E-10	1.025E-07
8	antigen processing and presentation of endogenous peptide antigen via MHC class I	6/29	9.379E-10	2.233E-07
9	antigen processing and presentation of endogenous peptide antigen	6/32	1.774E-09	3.754E-07
10	antigen processing and presentation of endogenous antigen	6/34	2.617E-09	4.985E-07

**Table 3:** Top 10 enriched process networks of regulated proteins in culture supernatants.

No.	Processes	Regulated	p-value	FDR
1	Cytoskeleton_Intermediate filaments	7/81	1.433E-06	1.218E-04
2	Protein folding_Response to unfolded proteins	5/69	1.207E-04	5.129E-03
3	Immune response_Phagosome in antigen presentation	7/243	1.555E-03	4.405E-02
4	Apoptosis_Apoptotic nucleus	5/159	5.271E-03	1.021E-01
5	Inflammation_NK cell cytotoxicity	5/164	6.003E-03	1.021E-01
6	Proliferation_Negative regulation of cell proliferation	5/184	9.658E-03	1.352E-01
7	Signal transduction_Androgen receptor nuclear signaling	4/126	1.216E-02	1.352E-01
8	Immune response_Antigen presentation	5/197	1.272E-02	1.352E-01
9	Cell adhesion_Cell-matrix interactions	5/211	1.671E-02	1.578E-01
10	Inflammation_IL-13 signaling pathway	3/91	2.680E-02	2.278E-01

**Table 4:** Number of differentially regulated secreted bacterial proteins in culture supernatants by each species.

Species	tissue + biofilm	overlap (up)	overlap (down)	biofilm
*A. oris*	0	0	0	0
*A. actinomycetemcomitans*	0	0	0	0
*C. rectus*	0	0	0	0
*F. nucleatum*	115	1	0	0
*P. gingivalis*	0	0	0	0
*P. intermedia*	8	1	0	0
*S. anginosus*	1	0	3	0
*S. oralis*	2	0	6	5
*T. forsythia*	0	0	0	0
*T. denticola*	0	0	0	2
*V. dispar*	0	0	0	8

Regulation trends of the quantified proteins from culture supernatants are listed in the table based on whether they were uniquely identified in tissue + biofilm, biofilm alone, or based on their up- or down-regulation trend in the overlap group. The regulation trends were considered using peptide counting methods compare tissue + biofilm group to the biofilm group alone.

**Table 5:** Number identified biofilm lysates proteins from each species.

Species	tissue + biofilm	Overlap	biofilm
*A. oris*	58	5	1
*A. actinomycetemcomitans*	8	13	2
*C. rectus*	45	79	3
*F. nucleatum*	41	791	125
*P. gingivilas*	5	172	55
*P. intermdia*	12	341	176
*S. anginosus*	25	88	10
*S. oralis*	202	252	3
*T. forsythia*	1	1	0
*T. denticola*	75	9	2
*V. dispar*	1	68	371
*H. sapiens*	235	67	21
sum	708	1886	769

Proteins identified from biofilm lysates were listed on the table based on whether they were uniquely identified in tissue + biofilm, biofilm alone, or overlapping proteins groups (identified in both groups).

**Table 6:** Number of differentially regulated proteins in biofilm lysate by each species.

Species	tissue + biofilm	overlap (up)	overlap (down)	biofilm
*A. oris*	39	5	0	0
*A. actinomycetemcomitans*	2	7	0	2
*C. rectus*	15	19	1	1
*F. nucleatum*	20	30	44	32
*P. gingivalis*	2	7	12	13
*P. intermedia*	5	27	79	66
*S. anginosus*	16	19	3	5
*S. oralis*	121	117	1	0
*T. forsythia*	1	0	0	0
*T. denticola*	58	8	0	0
*V. dispar*	0	0	49	255

Regulation trends of quantified proteins from biofilm lysates were listed on the table based on whether they were uniquely identified in tissue + biofilm, biofilm alone, or based on their up- or down-regulation trend in the overlap group. The regulation trends were considered using peptide counting methods compare tissue + biofilm group to the biofilm group alone.

## References

[b1] Darveau R. P. Periodontitis: a polymicrobial disruption of host homeostasis. Nat Rev Microbiol 8, 481–490, doi: 10.1038/nrmicro2337 (2010). 20514045

[b2] Page R. C. & Kornman K. S. The pathogenesis of human periodontitis: an introduction. Periodontol 2000 14, 9–11 (1997). 9567963 10.1111/j.1600-0757.1997.tb00189.x

[b3] Aas J. A., Paster B. J., Stokes L. N., Olsen I. & Dewhirst F. E. Defining the normal bacterial flora of the oral cavity. J Clin Microbiol 43, 5721–5732, doi: 10.1128/JCM.43.11.5721-5732.2005 (2005). 16272510 PMC1287824

[b4] Berezow A. B. & Darveau R. P. Microbial shift and periodontitis. Periodontol 2000 55, 36–47, doi: 10.1111/j.1600-0757.2010.00350.x (2011). 21134227 PMC3058494

[b5] Ramsey M. M., Rumbaugh K. P. & Whiteley M. Metabolite cross-feeding enhances virulence in a model polymicrobial infection. PLoS pathog 7, e1002012, doi: 10.1371/journal.ppat.1002012 (2011). 21483753 PMC3069116

[b6] Bachtiar E. W. *et al.* AI-2 of Aggregatibacter actinomycetemcomitans inhibits Candida albicans biofilm formation. Front Cellul Infect Microbiol 4, 94, doi: 10.3389/fcimb.2014.00094 (2014). PMC4104835 25101248

[b7] Jenkinson H. F. & Lamont R. J. Oral microbial communities in sickness and in health. Trends microbiol 13, 589–595, doi: 10.1016/j.tim.2005.09.006 (2005). 16214341

[b8] Hajishengallis G. Immunomicrobial pathogenesis of periodontitis: keystones, pathobionts, and host response. Trends immunol 35, 3–11, doi: 10.1016/j.it.2013.09.001 (2014). 24269668 PMC3947349

[b9] Hajishengallis G. & Lamont R. J. Beyond the red complex and into more complexity: the polymicrobial synergy and dysbiosis (PSD) model of periodontal disease etiology. Mol Oral Microbiol 27, 409–419, doi: 10.1111/j.2041-1014.2012.00663.x (2012). 23134607 PMC3653317

[b10] Seguier S., Godeau G. & Brousse N. Immunohistological and morphometric analysis of intra-epithelial lymphocytes and Langerhans cells in healthy and diseased human gingival tissues. Arch Oral Biol 45, 441–452 (2000). 10775673 10.1016/s0003-9969(00)00018-2

[b11] Locke M., Hyland P. L., Irwin C. R. & Mackenzie I. C. Modulation of gingival epithelial phenotypes by interactions with regionally defined populations of fibroblasts. J Periodontal Res 43, 279–289, doi: 10.1111/j.1600-0765.2007.01028.x (2008). 18447855

[b12] Belibasakis G. N. & Bostanci N. The RANKL-OPG system in clinical periodontology. J Clin Periodontol 39, 239–248, doi: 10.1111/j.1600-051X.2011.01810.x (2012). 22092994

[b13] Bao K., Papadimitropoulos A., Akgul B., Belibasakis G. N. & Bostanci N. Establishment of an oral infection model resembling the periodontal pocket in a perfusion bioreactor system. Virulence, 0, doi: 10.4161/21505594.2014.978721 (2015). PMC4601317 25587671

[b14] Bostanci N. *et al.* Label-free quantitative proteomics reveals differentially regulated proteins in experimental gingivitis. J Proteome Res, doi: 10.1021/pr300761e (2012). 23244068

[b15] Bostanci N. *et al.* Secretome of gingival epithelium in response to subgingival biofilms. Mol Oral Microbiol, doi: 10.1111/omi.12096 (2015). 25787257

[b16] Hendrickson E. L. *et al.* Proteomics of Fusobacterium nucleatum within a model developing oral microbial community. MicrobiologyOpen 3, 729–751, doi: 10.1002/mbo3.204 (2014). 25155235 PMC4234264

[b17] Bao K., Bostanci N., Selevsek N., Thurnheer T. & Belibasakis G. N. Quantitative proteomics reveal distinct protein regulations caused by Aggregatibacter actinomycetemcomitans within subgingival biofilms. PloS One 10, e0119222, doi: 10.1371/journal.pone.0119222 (2015). 25756960 PMC4355292

[b18] Bessarabova M., Ishkin A., JeBailey L., Nikolskaya T. & Nikolsky Y. Knowledge-based analysis of proteomics data. BMC Bioinformatics 13 **Suppl 16**, S13, doi: 10.1186/1471-2105-13-S16-S13 (2012). 23176192 PMC3489533

[b19] Moharamzadeh K. *et al.* Tissue-engineered oral mucosa. J Dent Res 91, 642–650, doi: 10.1177/0022034511435702 (2012). 22266525

[b20] Bao K., Akguel B. & Bostanci N. Establishment and Characterization of Immortalized Gingival Epithelial and Fibroblastic Cell Lines for the Development of Organotypic Cultures. Cells Tissues Organs, doi: 10.1159/000363694 (2014). 25471635

[b21] Thurnheer T. & Belibasakis G. N. Integration of non-oral bacteria into *in vitro* oral biofilms. Virulence, 0, doi: 10.4161/21505594.2014.967608 (2014). PMC4601515 25483866

[b22] Diaz P. I. *et al.* Synergistic interaction between Candida albicans and commensal oral streptococci in a novel *in vitro* mucosal model. Infect Immun 80, 620–632, doi: 10.1128/IAI.05896-11 (2012). 22104105 PMC3264323

[b23] Hajishengallis G. & Lambris J. D. Microbial manipulation of receptor crosstalk in innate immunity. Nat. Rev. Immunol. 11, 187–200, doi: 10.1038/nri2918 (2011). 21350579 PMC3077082

[b24] Belibasakis G. N., Thurnheer T. & Bostanci N. Interleukin-8 responses of multi-layer gingival epithelia to subgingival biofilms: role of the “red complex” species. PloS One 8, e81581, doi: 10.1371/journal.pone.0081581 (2013). 24339946 PMC3858256

[b25] Belibasakis G. N., Guggenheim B. & Bostanci N. Down-regulation of NLRP3 inflammasome in gingival fibroblasts by subgingival biofilms: involvement of Porphyromonas gingivalis. Innate Immun. 19, 3–9, doi: 10.1177/1753425912444767 (2013). 22522430

[b26] Bostanci N., Meier A., Guggenheim B. & Belibasakis G. N. Regulation of NLRP3 and AIM2 inflammasome gene expression levels in gingival fibroblasts by oral biofilms. Cell Immunol 270, 88–93, doi: 10.1016/j.cellimm.2011.04.002 (2011). 21550598

[b27] Bostanci N. *et al.* Application of label-free absolute quantitative proteomics in human gingival crevicular fluid by LC/MS E (gingival exudatome). J Proteome Res 9, 2191–2199, doi: 10.1021/pr900941z (2010). 20205380

[b28] Belibasakis G. N., Bao K. & Bostanci N. Transcriptional profiling of human gingival fibroblasts in response to multi-species *in vitro* subgingival biofilms. Mol Oral Microbiol, doi: 10.1111/omi.12053 (2014). 24758474

[b29] Nanci A. & Bosshardt D. D. Structure of periodontal tissues in health and disease. Periodontol 2000 40, 11–28, doi: 10.1111/j.1600-0757.2005.00141.x (2006). 16398683

[b30] Okinaga T., Kasai H., Tsujisawa T. & Nishihara T. Role of caspases in cleavage of lamin A/C and PARP during apoptosis in macrophages infected with a periodontopathic bacterium. J Med Microbiol 56, 1399–1404, doi: 10.1099/jmm.0.47193-0 (2007). 17893180

[b31] Li W. *et al.* Decreased bone formation and osteopenia in lamin a/c-deficient mice. PloS One 6, e19313, doi: 10.1371/journal.pone.0019313 (2011). 21547077 PMC3081846

[b32] Ohlrich E. J., Cullinan M. P. & Seymour G. J. The immunopathogenesis of periodontal disease. Aust Dent J 54 **Suppl 1**, S2–10, doi: 10.1111/j.1834-7819.2009.01139.x (2009). 19737265

[b33] Bomberger J. M. *et al.* Pseudomonas aeruginosa Cif protein enhances the ubiquitination and proteasomal degradation of the transporter associated with antigen processing (TAP) and reduces major histocompatibility complex (MHC) class I antigen presentation. J Biol Chem 289, 152–162, doi: 10.1074/jbc.M113.459271 (2014). 24247241 PMC3879540

[b34] Van Dyken S. J. & Locksley R. M. Interleukin-4- and interleukin-13-mediated alternatively activated macrophages: roles in homeostasis and disease. Annu Rev Immunol 31, 317–343, doi: 10.1146/annurev-immunol-032712-095906 (2013). 23298208 PMC3606684

[b35] Sorsa T. *et al.* Matrix metalloproteinases: contribution to pathogenesis, diagnosis and treatment of periodontal inflammation. Ann Med 38, 306–321, doi: 10.1080/07853890600800103 (2006). 16938801

[b36] Peyret-Lacombe A., Brunel G., Watts M., Charveron M. & Duplan H. TLR2 sensing of F. nucleatum and S. sanguinis distinctly triggered gingival innate response. Cytokine 46, 201–210, doi: 10.1016/j.cyto.2009.01.006 (2009). 19299164

[b37] Eskan M. A., Hajishengallis G. & Kinane D. F. Differential activation of human gingival epithelial cells and monocytes by Porphyromonas gingivalis fimbriae. Infect Immun 75, 892–898, doi: 10.1128/IAI.01604-06 (2007). 17118977 PMC1828485

[b38] Hatakeyama J. *et al.* Contrasting responses of human gingival and periodontal ligament fibroblasts to bacterial cell-surface components through the CD14/Toll-like receptor system. Oral Microbiol Immunol 18, 14–23 (2003). 12588454 10.1034/j.1399-302x.2003.180103.x

[b39] Belibasakis G. N. & Guggenheim B. Induction of prostaglandin E(2) and interleukin-6 in gingival fibroblasts by oral biofilms. FEMS Immnol Med Mic 63, 381–386, doi: 10.1111/j.1574-695X.2011.00863.x (2011). 22092565

[b40] Belibasakis G. N., Meier A., Guggenheim B. & Bostanci N. The RANKL-OPG system is differentially regulated by supragingival and subgingival biofilm supernatants. Cytokine 55, 98–103, doi: 10.1016/j.cyto.2011.03.009 (2011). 21474331

[b41] Marsh P. D. Are dental diseases examples of ecological catastrophes? Microbiology 149, 279–294 (2003). 12624191 10.1099/mic.0.26082-0

[b42] Takahashi N. Microbial ecosystem in the oral cavity: Metabolic diversity in an ecological niche and its relationship with oral diseases. Int Congr Ser 1284, 103–112 (2005).

[b43] Kolenbrander P. E. *et al.* Bacterial interactions and successions during plaque development. Periodontol 2000 42, 47–79, doi: 10.1111/j.1600-0757.2006.00187.x (2006). 16930306

[b44] Han Y. W. *et al.* Interactions between periodontal bacteria and human oral epithelial cells: Fusobacterium nucleatum adheres to and invades epithelial cells. Infect Immun 68, 3140–3146 (2000). 10816455 10.1128/iai.68.6.3140-3146.2000 PMC97547

[b45] Dabija-Wolter G. *et al.* Fusobacterium nucleatum enters normal human oral fibroblasts *in vitro*. J Periodontol 80, 1174–1183, doi: 10.1902/jop.2009.090051 (2009). 19563299

[b46] Abe K. Butyric acid induces apoptosis in both human monocytes and lymphocytes equivalently. J Oral Sci 54, 7–14 (2012). 22466881 10.2334/josnusd.54.7

[b47] Saito A. *et al.* Fusobacterium nucleatum enhances invasion of human gingival epithelial and aortic endothelial cells by Porphyromonas gingivalis. FEMS Immunol Med Microbiol 54, 349–355, doi: 10.1111/j.1574-695X.2008.00481.x (2008). 19049647

[b48] Metzger Z., Blasbalg J., Dotan M. & Weiss E. I. Enhanced attachment of Porphyromonas gingivalis to human fibroblasts mediated by Fusobacterium nucleatum. J Endod 35, 82–85, doi: 10.1016/j.joen.2008.10.011 (2009). 19084131

[b49] Periasamy S. & Kolenbrander P. E. Mutualistic biofilm communities develop with Porphyromonas gingivalis and initial, early, and late colonizers of enamel. J Bacteriol 191, 6804–6811, doi: 10.1128/JB.01006-09 (2009). 19749049 PMC2772475

[b50] Rickard A. H. *et al.* Autoinducer 2: a concentration-dependent signal for mutualistic bacterial biofilm growth. Mol Microbiol 60, 1446–1456, doi: 10.1111/j.1365-2958.2006.05202.x (2006). 16796680

[b51] Ammann T. W., Belibasakis G. N. & Thurnheer T. Impact of early colonizers on *in vitro* subgingival biofilm formation. PloS One 8, e83090, doi: 10.1371/journal.pone.0083090 (2013). 24340084 PMC3855599

[b52] Teles F. R. *et al.* Early microbial succession in redeveloping dental biofilms in periodontal health and disease. J Periodontal Res 47, 95–104, doi: 10.1111/j.1600-0765.2011.01409.x (2012). 21895662 PMC3253172

[b53] Arce R. M. *et al.* Increased TLR4 expression in murine placentas after oral infection with periodontal pathogens. Placenta 30, 156–162, doi: 10.1016/j.placenta.2008.11.017 (2009). 19101032 PMC2656361

[b54] Gaetti-Jardim E. Jr. *et al.* Subgingival microbiota from Cebus apella (capuchin monkey) with different periodontal conditions. Anaerobe 18, 263–269, doi: 10.1016/j.anaerobe.2012.02.002 (2012). 22710412

[b55] Visser M. B. & Pollitt C. C. The timeline of metalloprotease events during oligofructose induced equine laminitis development. Equine Vet J 44, 88–93, doi: 10.1111/j.2042-3306.2011.00393.x (2012). 21696433

[b56] Nogales E., Downing K. H., Amos L. A. & Lowe J. Tubulin and FtsZ form a distinct family of GTPases. Nat Struct Biol 5, 451–458 (1998). 9628483 10.1038/nsb0698-451

[b57] Adams D. W. & Errington J. Bacterial cell division: assembly, maintenance and disassembly of the Z ring. Nature Rev Microbiol 7, 642–653, doi: 10.1038/nrmicro2198 (2009). 19680248

[b58] Robinson M. D., McCarthy D. J. & Smyth G. K. edgeR: a Bioconductor package for differential expression analysis of digital gene expression data. Bioinformatics 26, 139–140, doi: 10.1093/bioinformatics/btp616 (2010). 19910308 PMC2796818

[b59] Deng W., Wang Y., Liu Z., Cheng H. & Xue Y. HemI: a toolkit for illustrating heatmaps. PloS One 9, e111988, doi: 10.1371/journal.pone.0111988 (2014). 25372567 PMC4221433

[b60] Supek F., Bosnjak M., Skunca N. & Smuc T. REVIGO summarizes and visualizes long lists of gene ontology terms. PloS One 6, e21800, doi: 10.1371/journal.pone.0021800 (2011). 21789182 PMC3138752

