# Supplementary material for: Proteomic profiling of host-biofilm interactions in an oral infection model resembling the periodontal pocket
Source: Sci Rep. 2015 Nov 3;5:15999. doi: 10.1038/srep15999 (PMC4630604; doi:10.1038/srep15999)
Supplement: Supplementary Information [file srep15999-s1.pdf]

Supplementary Figures for:

Proteomic profiling of host-biofilm interactions in an oral infection model resembling the periodontal pocket

Kai Bao[1], Georgios N. Belibasakis[2], Nathalie Selevsek[3], Jonas Grossmann[3], Nagihan Bostanci[1*]

[1] Oral Translational Research, Institute for Oral Biology, Center of Dental Medicine, University of Zürich, Zürich, Switzerland

[2] Oral Microbiology and Immunology, Institute for Oral Biology, Center of Dental Medicine, University of Zürich, Zürich, Switzerland

[3] Functional Genomics Center Zürich, University of Zürich, Zürich, Switzerland

* Corresponding author

E-mail: Nagihan.Bostanci@zzm.uzh.ch (NB)

a)

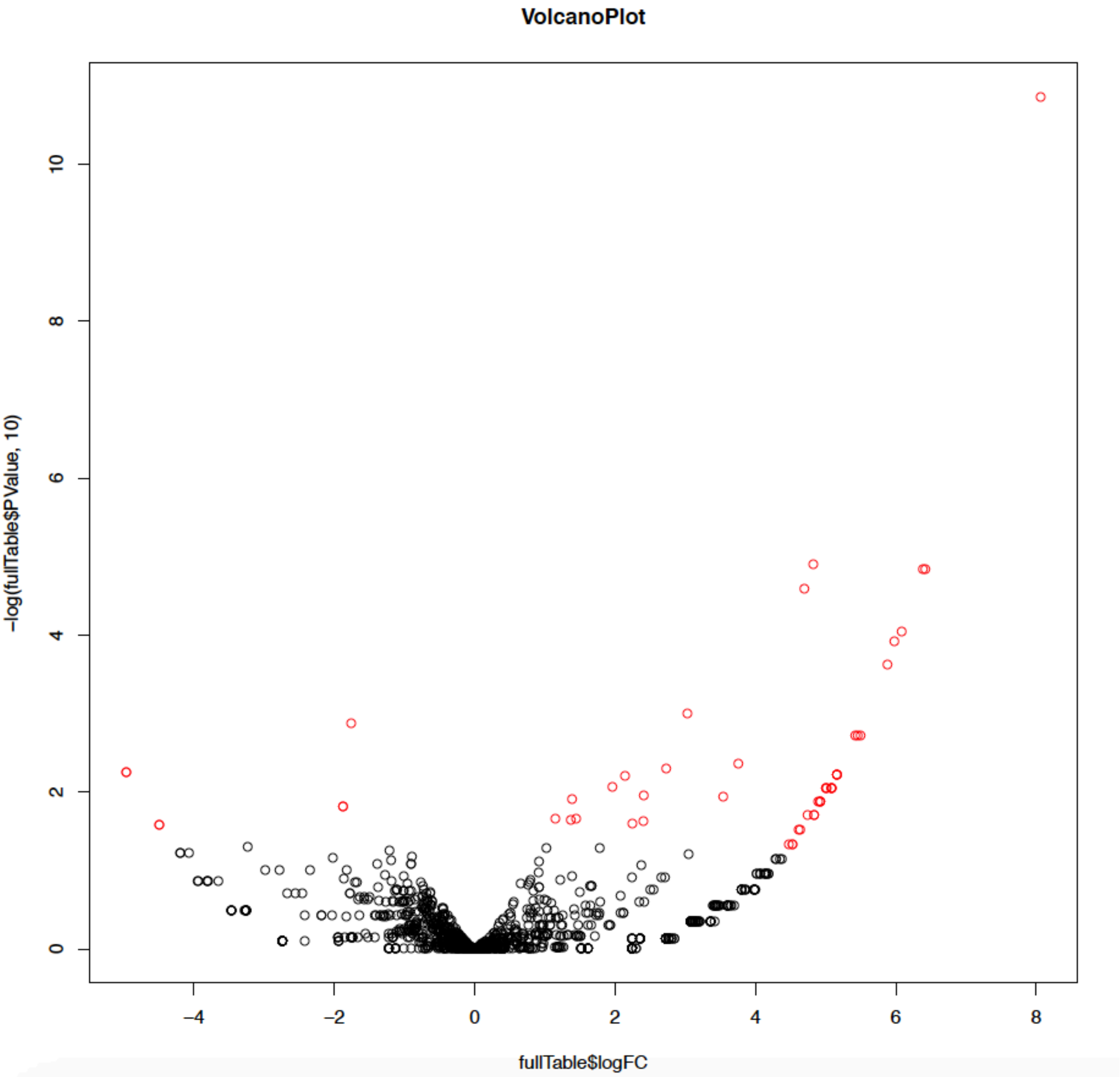
VolcanoPlot
−log(fullTable$PValue, 10)
fullTable$logFC
0
2
4
6
8
10
−4
−2
0
2
4
6
8

b)

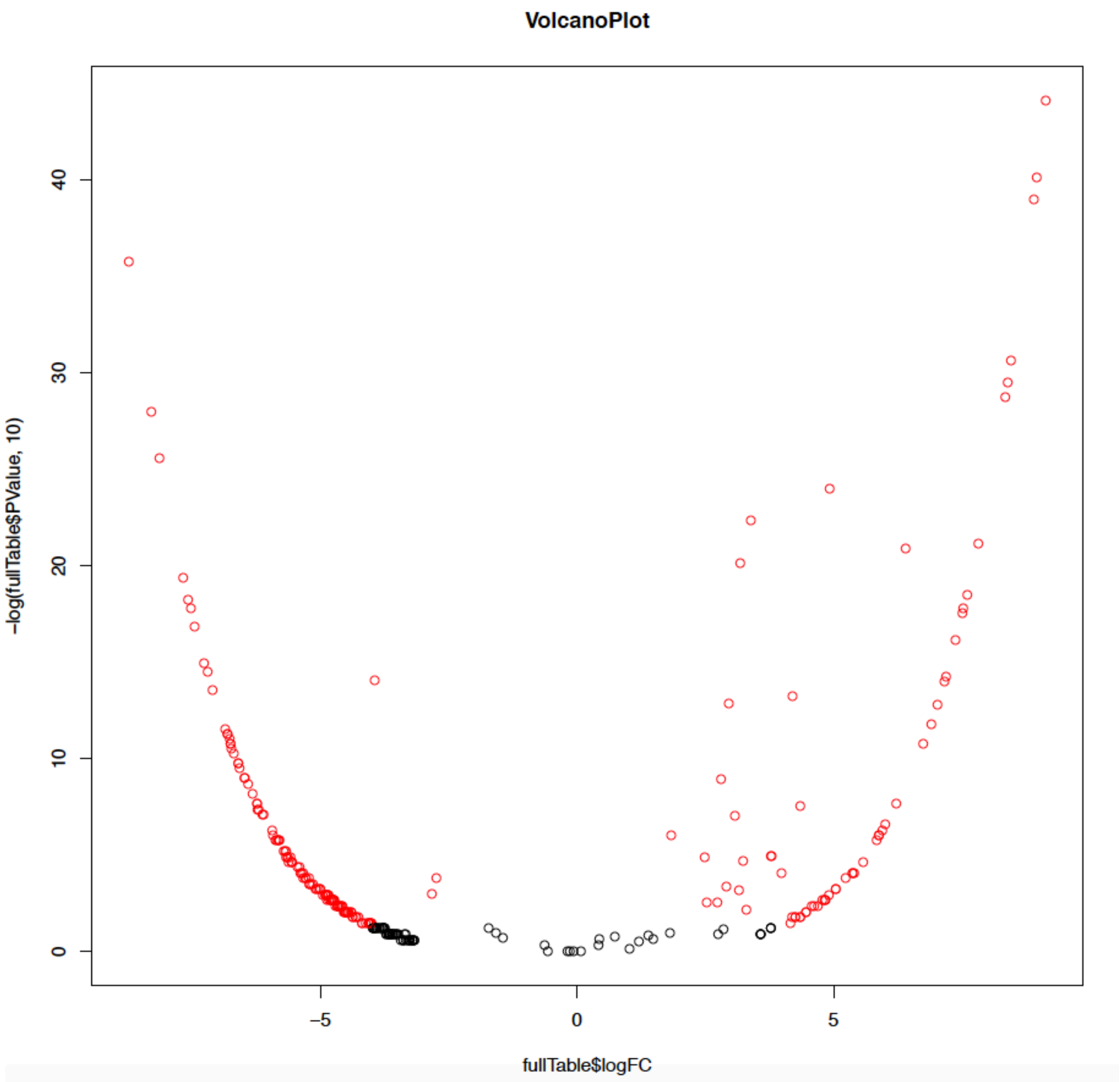
VolcanoPlot
-log(fullTable$PValue, 10)
0
10
20
30
40
−5
0
5
fullTable$logFC

c)

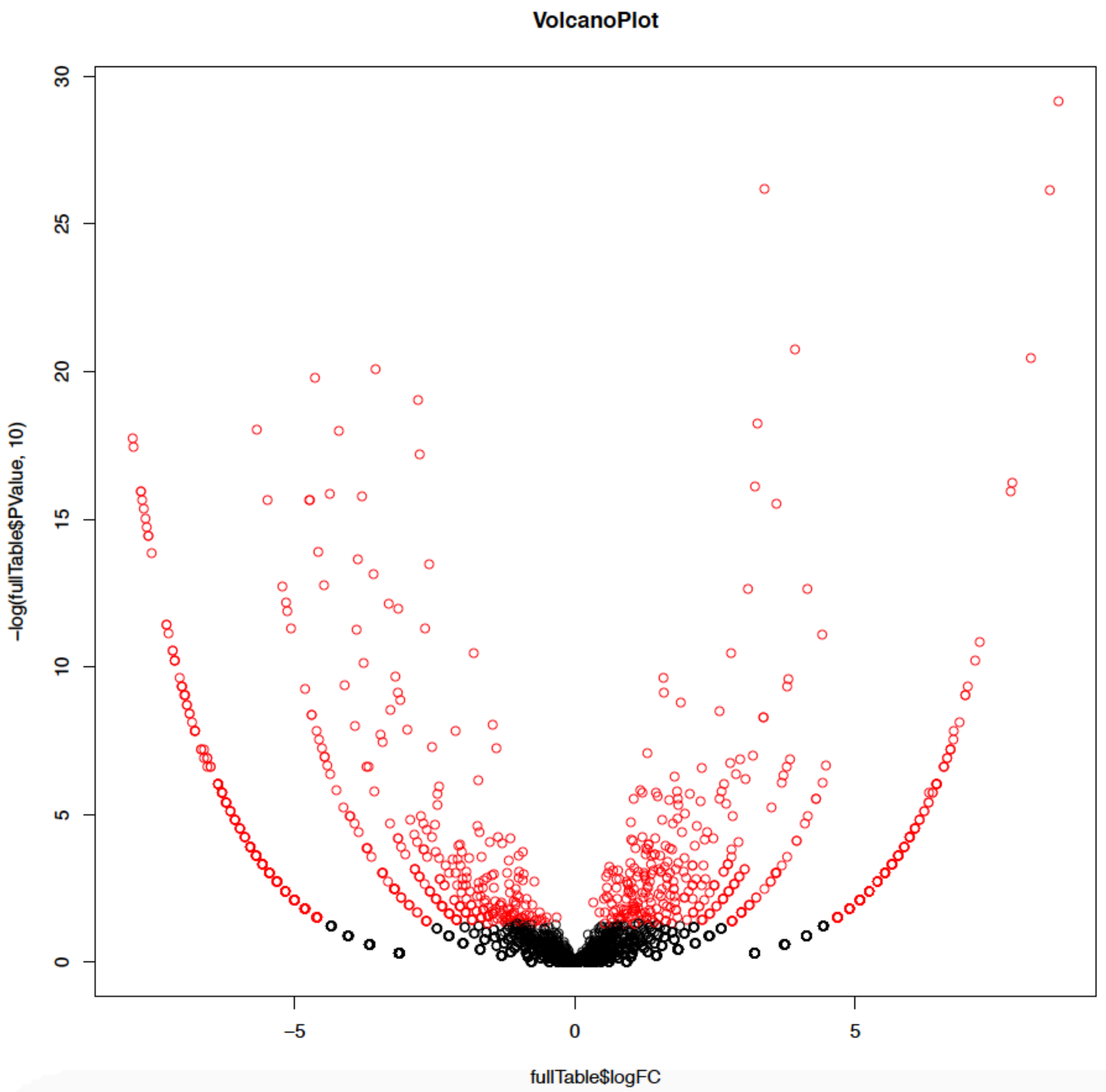

Supplementary Information Figure S1: Regulated proteins in different conditions. Shown are volcano plots of a) regulated human proteins among the identified human proteins in bioreactor medium; b) regulated bacterial proteins among the identified bacterial proteins in bioreactor medium; c) regulated bacterial proteins among the identified human proteins in biofilm lysates. Plots represents regulated proteins were showed in red colour, while unregulated proteins were shown in black colour. y-axis - negative log 10 of p value; x-axis - log2-transformed fold-change; red dots - proteins with significant p value; black dots - proteins sets with non-significant p value. Regulated proteins were determined by LogFC $P < 0.05$ by R platform use edgeR package.

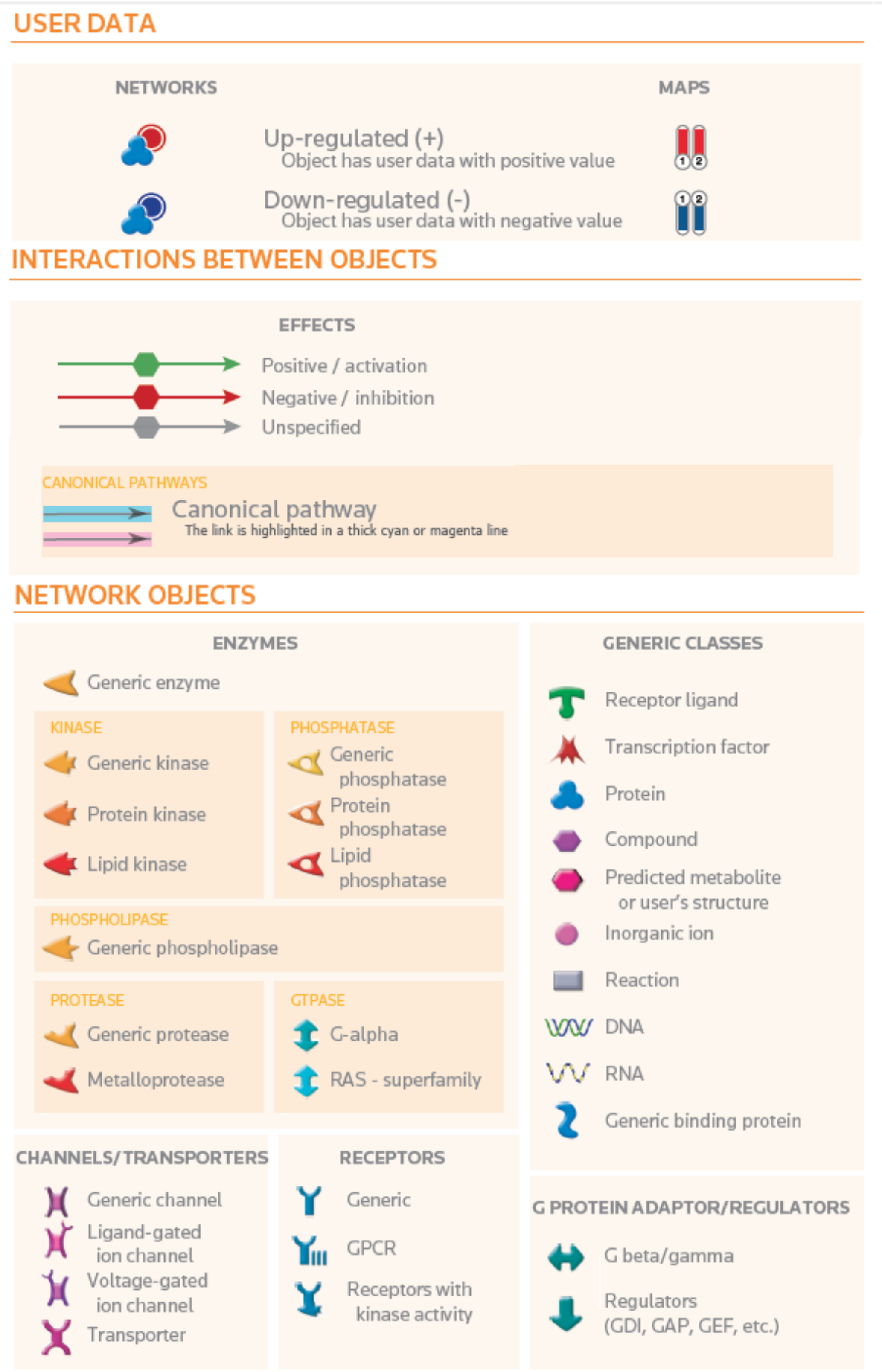

Supplementary Information Figure S2: **Detailed symbol legend of network analysis**
